# Svetlana a supervised segmentation classifier for Napari

**DOI:** 10.1038/s41598-024-60916-8

**Published:** 2024-05-21

**Authors:** Clément Cazorla, Renaud Morin, Pierre Weiss

**Affiliations:** 1 Institut de Mathématiques de Toulouse (IMT), Université de Toulouse, Toulouse, France; 2 Institut de Recherche en Informatique de Toulouse (IRIT), Centre de Biologie Intégrative (CBI), Laboratoire de biologie Moléculaire, Cellulaire et du Développement (MCD), Université de Toulouse, CNRS, Toulouse, France; 3 Imactiv-3D. Centre Pierre Potier, 1 place Pierre Potier, 31100 Toulouse, France

**Keywords:** Software, Segmentation, Classification, Convolutional neural networks, Biomedical imaging, Image analysis, Microscopy, Efficient AI, Image processing, Machine learning, Software

## Abstract

We present Svetlana (SuperVised sEgmenTation cLAssifier for NapAri), an open-source Napari plugin dedicated to the manual or automatic classification of segmentation results. A few recent software tools have made it possible to automatically segment complex 2D and 3D objects such as cells in biology with unrivaled performance. However, the subsequent analysis of the results is oftentimes inaccessible to non-specialists. The Svetlana plugin aims at going one step further, by allowing end-users to label the segmented objects and to pick, train and run arbitrary neural network classifiers. The resulting network can then be used for the quantitative analysis of biophysical phenoma. We showcase its performance through challenging problems in 2D and 3D and provide a comprehensive discussion on its strengths and limits.

## Introduction

Recent years have witnessed spectacular progress in biological imaging. We can think of the improvement and accessibility of high-resolution microscopes, the explosion in storage and computing resources, and the advances in artificial intelligence. This offers exciting prospects for better understanding life. These advances however hinge on the ability to automatically analyze large volumes of data and, in particular, to segment and classify biological structures.

Specialized tools have emerged. For instance, the HoVer-Net^1^ yields excellent performance for the quantification of histopathology images stained with specific compounds. Unfortunately, even though it effectively addresses an important and difficult issue, its adaptation to different datasets (imaging modality, staining, type of tissues, type of classification) is far from being obvious. To the best of our knowledge, there currently does not exist a general purpose classification software tools that could be used for arbitrary studies.

The situation is quite different for segmentation tasks. This is the result of concomitant facts including advances in machine learning, the creation of open training databases and the development of ergonomic open-source software packages. Carefully designed neural networks architectures provide unprecedented segmentation results. They make it possible to avoid setting hyper-parameters which are often hard to tune and interpret. Examples of powerful and popular tools for segmentation in biology include Ilastik^2^, CellPose^3^, Omnipose^4^, StarDist^5^, ZeroCostDL4Miic^6^, Deep-ImageJ^7^ or Sketchpose^8^. Their performance and ergonomics continue to improve at a fast pace.

**Our motivation** Segmentation masks—as good as they are—are rarely directly exploitable to answer biological questions. In particular, it is often necessary to classify the detected objects in order to perform statistical analyses that give a concrete meaning to the results. The precise cells boundary delineation can even be less scientifically significant than the quantification of its phenotypic characteristics. Despite the significant benefits of these segmentation tools, a difficult part of the analysis therefore remains inaccessible to most users.

**Our contribution** The goal of this work is to continue filling the gap between methodological advances and end-users, by providing a convenient software tool for the classification of segmentation results with a minimum amount of manual annotation.

## Plugin description

**Figure 1:**
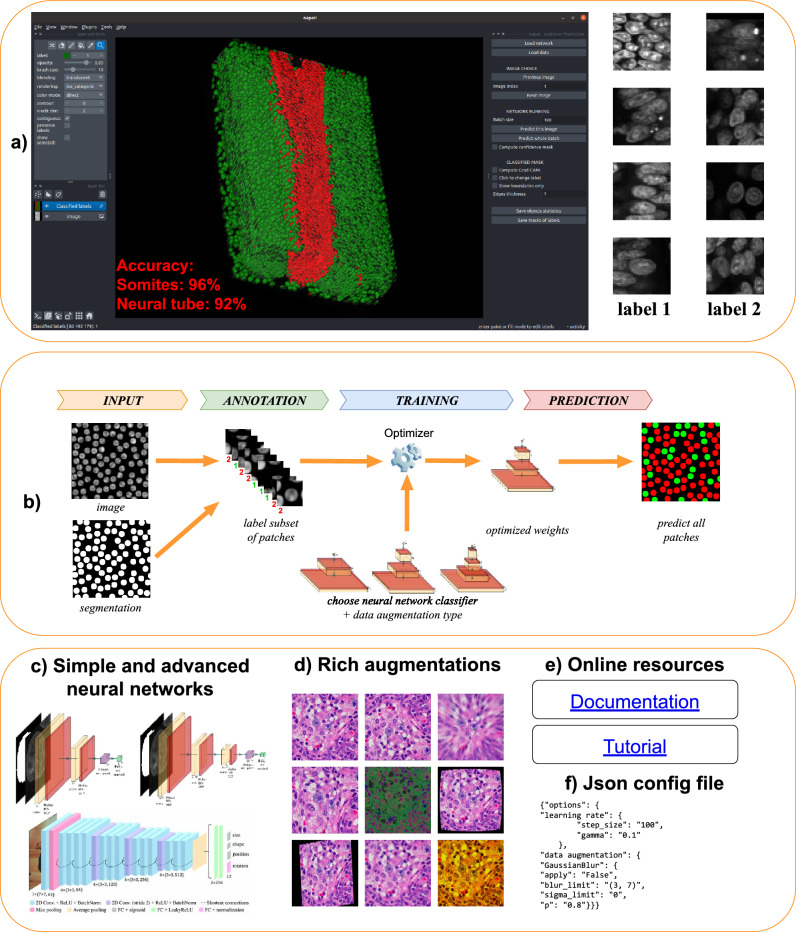
A schematic overview of the Svetlana plugin. (**a**) A screenshot of the plugin in action. In this example, Svetlana is able to separate the mesorderm (in green) and the neural tube (in red) nuclei of a quail embryo^9^ with a high accuracy after just a few clicks. (**b**) Overview of the Svetlana’s three-step pipeline: Annotation, Training and Prediction. Given pairs of images and segmentation masks, the user labels a few connected components. This set is then used to train a neural network classifier. Once trained, it can be used to classify one or multiple segmented images. (**c**) Svetlana offers many neural network architectures with increasing complexity. The minimalist architectures can be trained faster and are usually enough to lead to high classification accuracies. (**d**) The training can be enriched with a large variety of image augmentation techniques available in Albumentations^10^. (**e**) Online resources are available to assist the user. (**f**) All experiments can be fine tuned and reproduced using a JSON configuration file.

We designed a user-friendly plugin called Svetlana. This name is an acronym for SuperVised sEgmenTation cLAssifier for NapAri^11^. At the end of a simple annotation and training process, the user is provided with a neural network that automatically classifies large collections of segmented images. For instance, we show in Fig. 1a how it can automatically count and distinguish cells belonging to two different types in a developing quail embryo after just a few clicks.

Our aim was to create a plugin that fulfills the majority of requirements for image analysis platforms. It’s versatile, handling 2D, 3D, grayscale or multi-spectral images, whether individual or in large collections. It is well documented with a rather simple installation procedure, see the hyperlinks in Fig. 1e.

The annotation module offers various labeling modes, enabling the annotation of thousands of 2D or 3D objects in minutes. The training module supports user-provided architectures as well as popular neural network architectures, see Fig. 1c. We have also designed minimalist neural network, ideal for simple classification tasks with limited annotations.

Additionally, the plugin includes a wide range of image augmentation techniques, see Fig. 1d. These techniques enhance the classifier stability across different image acquisition protocols and promote useful features such as rotation invariance.

Understanding how the neural network makes decisions can be crucial for some applications, such as deciphering biophysical phenomena or avoiding confounding variables. To this end, we integrated a network interpretation module named Grad-CAM^12^. Augmentation techniques can also facilitate interpretation through “augmentation” studies. By adding or removing augmentation types at training time and evaluating the classification performance, the user can see which features are essential. For instance if changing the image colors randomly during the training implies a performance drop for the classification, it means that a color is probably important to distinguish between cell populations.

Overall, Svetlana offers a complete solution to design robust, accurate and interpretable neural-network-based classifiers.

### Workflow of Svetlana

The principle of Svetlana is displayed in Fig. 1b. It relies on an external segmentation module which outputs a segmentation mask. Svetlana then takes *the images to be labeled and the segmentation masks* as inputs. It is separated in three different modules:

*Annotation*: This module allows the user to label some connected components of the segmentation masks. For simple classification tasks in 2D or 3D, we could label more than 1000 connected components in about 15 min.
*Training*: This module allows the user to pick an arbitrary PyTorch^13^ neural network architecture (possibly pre-trained) and to further train it with the annotations generated by the previous module. A few seconds or minutes are usually enough to provide high quality classification results (see Table 1).
*Prediction*: This module uses the trained network to classify one or many segmentation masks.

The outputs of the plugin are: *a set of manually annotated patches, a trained neural network and prediction masks*. The results are stored in files under widely accessible formats for the forthcoming analyses.

### Requirements

We decided to integrate Svetlana in the Napari environment^14^, since it offers a rich interface to manipulate images and a direct connection to PyTorch, a dominant artificial intelligence Python package. The first installation step is to install a proper Python environment. To do so, we advise the user to first install Anaconda, Mamba or Pip. Installing Svetlana can then be done simply by following the instructions on the installation page. It will be available in the plugin manager of Napari. Ideally, it should be used with a computer equipped with an Nvidia GPU (Graphical Processing Unit) for faster processing.

### Main processing steps

In this section, we describe the main steps to design an efficient classifier in Svetlana.

**Annotation** After organizing the images and segmentation masks in the right folders, the first step consists in annotating a few regions of interest (ROI). Various annotation modes are available: the ROI can be selected by clicking or picked randomly by the plugin. This works using both the 2D and 3D visualization. The label can be assigned by giving a number from 1 to 9, which is the maximum number of classes.

Depending on the complexity of the classification task, the number of ROI to label to generate a good classifier can vary from a few units to a few hundreds. It is hard to anticipate this. A possible solution is to start with few annotations and if the classifier is not accurate enough, additional annotations can be provided in a second stage. This active learning mecanism allows the user to rapidly generate a satisfactory training set.

**Choosing a CNN architecture** We designed minimalist convolutional neural network (CNN) architectures adpated to 2D or 3D images with arbitrary number of channels. They are defined by their width and depth, see Fig. 1c. In all our experiments, these architectures which contain few trainable weights proved to be sufficient to reach accurate classification results. We therefore recommend using these first. As illustrated in Table 2, the depth and width of the architecture yield minor differences in the final accuracy and a width of 32 and depth of 3 seems like a good compromise.

In addition, Svetlana offers a large number of popular pre-defined architectures in 2D (ResNet^15^, DenseNet^16^, AlexNet^17^ of various depths). They contain millions of parameters, which can be excessive for small dataset. Pre-trained networks can also be loaded and fine-tuned using Svetlana with additional data.

**Optimization routine** Svetlana uses Adam as an optimization algorithm^18^. It is widely accepted as one of the most versatile method for training neural networks. The main parameters are the number of iterations, the batch size, the learning rate and the momentum. They can all be set in the interface. Our experiments revealed that a step decay stabilizes the optimization process. If needed, additional options can be specified in the Json configuration file.

**Patch normalization** In most situations, we advise to rescale the patches in [0, 1] using the min-max scaling available in the interface. If the intensity plays an important role to distinguish cell types, other options are available.

**Data augmentation** A quite useful feature of Svetlana is the possibility to use data augmentation during the training step.

To avoid overloading the graphical interface, the augmentation possibilities are limited to vertical and horizontal flips and random rotations. It is however possible to use the much richer set of transformations available within the Albumentations library^10^ by setting the configuration file. The transformations are specified in the form of a dictionary, as indicated in the documentation.

**Loss functions** We recommend using the cross-entropy as a loss function even though other possibilities are offered for more advanced users.

**The batch mode** Svetlana can be used on single images or entire folders for the annotation, training and prediction plugins. It is therefore possible to build a large training dataset, possibly containing images from different modalities.

**Active learning / human-in-the-loop** The annotation interface offers the possibility to correct the predictions after training. The previous prediction mask is loaded as an overlay and the user can correct or complete his annotation, before restarting a training.

## Validation of the method

To showcase the usefulness of Svetlana, we performed three experiments on real biological problems.

### Quail embryo nuclei classification

The first problem is provided as a demo in the plugin. It does not have a particularly important biological relevance, but illustrates the main plugin features. We use a $624 \times 158 \times 232$ voxels crop of a large quail embryo image. It was taken with a two-photon microscope with a resolution of $0.68\times 0.68 \times 1$ µm by B. Bénazéraf^9^.

The cropped part contains two structures of interest:

- The neural tube: an axial tissue that will form the spinal cord.
- The somites: round structures located on either side of the neural tube that will form skeletal muscles and vertebrae.

The classification task we are interested in is to distinguish between nuclei within the neural tube and those within the mesoderm. They can be easily distinguished by visual inspection, allowing the user to rapidly annotate a few of them.

To classify the two cell types, we used the following protocol:

1. Segment the whole volume using the model Cyto2 in CellPose.
2. Annotate the 3D image using Svetlana (169 annotations in about 5 minutes).
3. Train a two-layer 3D convolutional network ($\approx$ 7 min).
4. Predict all the nuclei types in the image ($\approx$ 10 min).

We used patches of size $45\times 45 \times 45$ voxels from the complete 3D image for the prediction. The classification accuracy is more than 94% on both nuclear types, see Fig. 1a. Obtaining such a result using only 169 annotations out of 26, 214 is remarkable. Indeed, we have already shown in Fig. 4g, that a 2D random forest classifier was unable to accomplish this task, based on simple radiometric and morphometric features. We performed a similar experiment in 3D, and again, the classification were nearly random. We do not reproduce it in this paper for conciseness.

### Pathology dataset classification

**Figure 2:**
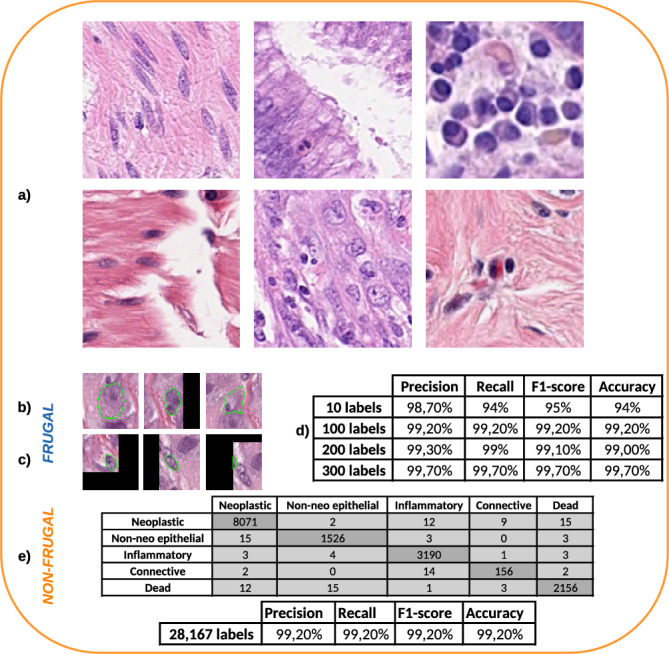
A histopathology images classification problem. (**a**) A subset of 6 images taken from PanNuke dataset. (**b**) and (**c**) Example of $137\times 137$ pixels training patches showing neoplastic and inflammatory cells respectively. The nucleus of interest has been circled in green for visualization. We can see that the two classes are difficult to distinguish visually for a non-expert. (**d**) Classification performance by training the model on increasingly large 2-class (neoplastic/inflammatory cells) small datasets. (**e**) Classification performance on a 5-class problem training with a large dataset.

In this section, we showcase that Svetlana’s minimalist neural networks can solve complex classification problems, using a public dataset called PanNuke^19^ (see Fig. 2a). The dataset comprises 481 visual fields, with 312 selected randomly from over 20,000 whole slide images sourced from various data sources, captured at different magnifications. It is composed of 205,343 nuclei categorized into 5 clinical classes (neoplastic, non-neo epithelial, inflammatory, connective, dead) from 19 different tissues. Other factors of variability, such as the diversity of patients from whom tissues are extracted, or the difficulty of obtaining reproducible tissues staining, make this type of dataset challenging to classify robustly.

We demonstrate Svetlana’s ability to produce accurate classification results for this type of application both for a small and a large dataset. We used a neural network of depth 3 and width 32 available in Svetlana. It is composed of 14,500 parameters. This is about 1000 times smaller than a Resnet18^15^, which is among the smallest classification models from the Torchvision library.

To quantify the classification results, we compute standard precision metrics. Let $n_i$ denote the number of elements in the *i*-th class. We compute metrics based on the numbers of True Positives ($TP_i$), False Positives ($FP_i$), False Negatives ($FN_i$) and True Negatives ($TN_i$) for each class. The metrics are then defined as follows:

$$\begin{aligned} \text {Precision}&= \frac{1}{N} \sum _{i=1}^{N} \left( \frac{n_i \times \text {TP}_i}{n_i \times \text {TP}_i + \text {FP}_i} \right) \\ \text {Recall}&= \frac{1}{N} \sum _{i=1}^{N} \left( \frac{n_i \times \text {TP}_i}{n_i \times \text {TP}_i + \text {FN}_i} \right) \\ \text {F1-Score}&= 2 \times \frac{\text {Precision} \times \text {Recall}}{\text {Precision} + \text {Recall}} \\ \text {Accuracy}&= \frac{1}{N} \sum _{i=1}^{N} \left( \frac{\text {TP}_i + \text {TN}_i}{\text {TP}_i + \text {FP}_i + \text {TN}_i + \text {FN}_i} \right) \end{aligned}$$

We assess Svetlana’s performance in two different scenarios. First, we trained our custom minimalist architecture on various subsets containing from 10 to 300 labels. For this experiment, we launch the training on two classes: the neoplastic and inflammatory cells. Figure 2b contains examples of neoplastic cells and Fig. 2c contain examples of inflammatory cells. Figure 2d demonstrates that with only 10 labels, we already reach a classification accuracy of about $94\%$. Increasing the dataset size to 300 yields high precision and accuracy for both classes ($99.70\%$).

In a second step, we trained Svetlana on a much larger 5-class dataset containing 28,167 nuclei extracted from PanNuke dataset as well as for the previous experiment. In Fig. 2e, we computed the confusion matrix for the 5 classes, as well as the weighted average precision, recall, F1-score and accuracy, on a validation set of 15,218 nuclei. Again, high accuracies can be reached with the minimalist CNN architectures that we designed.

Overall this experiment shows that the Svetlana plugin and the minimalist neural network architectures offer a solid companion for tackling complex classification tasks, whether in a data-limited or data-rich context.

### Osteoclasts classification

**Figure 3:**
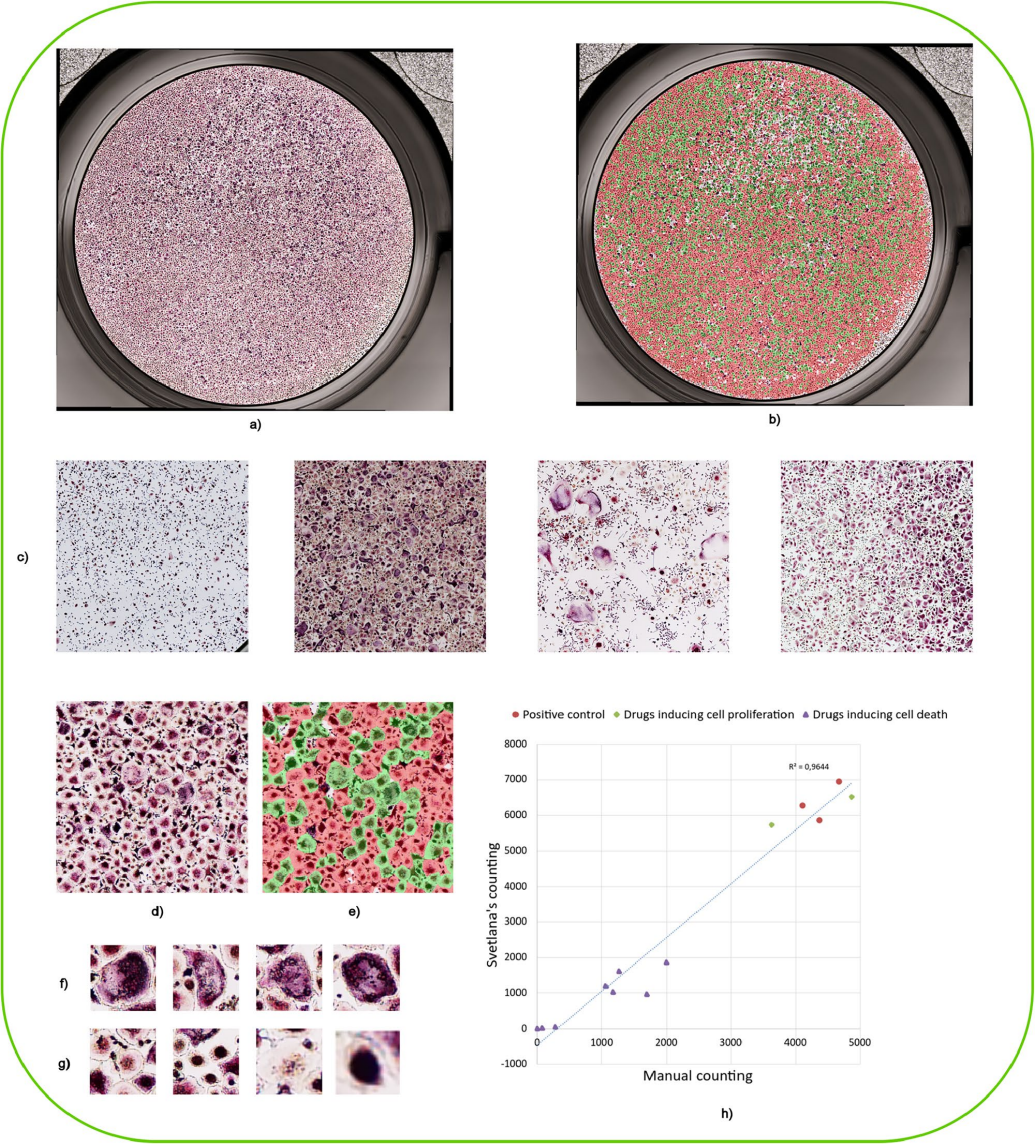
The osteoclasts classification—(**a,b**) An example of an entire $8000\times 8000$ pixels image and a classification result using Svetlana. The green spots correspond to activated osteoclasts. The image was provided by . Atlantic Bone Screen company (ABS) (**c**) $2000\times 2000$ pixels crops of four different images illustrating the diversity of the dataset. (**d,e**) $750\times 750$ pixels crop and its classification result after training a neural network with 600 annotations (out of 16,671). (**f**) Example of activated osteoclats. (**g**) Example of non-activated osteoclasts. (**h**) This graph shows the excellent correlation (96% coefficient of determination) between Svetlana’s counting and a human counting. Various conditions were tested. For the red dots, no drug was applied. For the green diamonds, drugs inducing cell proliferation were tested. For the purple triangles, drugs inducing cell death were introduced.

Segmentation in bright-field microscopy can be challenging due to low contrasts, out-of-focus blur and differences in staining across different samples for example. To illustrate that Svetlana overcomes these issues, we solved a cell classification problem used in medium throughput screening on osteoclasts. Osteoclasts are bone cells that go through different stages of differentiation which gives rise to a wide diversity of cell morphologies as shown in Fig. 3c. They are the subject of many studies (see Labour et al.^20^ for example) as they can be the cause of various diseases when dysfunctional. Indeed, a physiological unbalance between osteoclasts and osteoblasts (osteoclasts antagonistic cells), can lead to various forms of bone disease, such as osteoporosis. That is why it is so important to control their respective populations. In this experiment, patient osteoclasts are isolated and grown in culture. They are then distributed in wells (see Fig. 3a) and subjected to various molecules.

The Atlantic Bone Screen (ABS) company assesses the impact of each treatment by calculating the number of activated osteoclasts, the cell version able to perform bone resorption. Activated cells are differentiated from others as they are typically large, with a dark violet color and a large number of nuclei manifested as small dots inside the cell (polynucleate). A few activated osteoclasts are shown in Fig. 3f, while osteoclasts in different states are shown in Fig. 3g.

A well can contain up to 20,000 cells. The classification task is performed by specialists at ABS and includes a manual counting of activated osteoclasts. This expert approach is accurate, but time consuming for high cell density wells and may require the intervention of several experts to multiply the counts, in order to reduce inter-operator variability, and guarantee a high accuracy. Automating the classification process allows contract research organizations to save time on their projects, and analyze more data while reproducing their selection criteria.

The automatic classification task is really challenging due to a wide variety of image appearances, see Fig. 3c. These differences can be explained by the biological nature of the sample, but also by the diversity of patients, staining protocols, drug types and concentrations. To address this problem, we first improved the Cyto2 neural network available in CellPose2^21^ using its human-in-the-loop feature. This provided satisfactory segmentation results. We then trained our classifier on 5400 patches extracted from diverse images ($\approx$ 1 hour of manual labeling). We then turned to the prediction mode and applied the neural network classifier to the whole batch. Figure 3b shows a prediction on a whole well, while Fig. 3d and e show a crop and its automated classification respectively. The prediction on 63 images of size $8000\times 8000$ pixels took about 1.5 h, i.e. 1.4 min per image. This has to be compared to the 20 to 45 min taken by a specialist to process a portion of a single image by hand.

The graph in Fig. 3h shows that our classifier reaches coefficient of determination $R^{2}$ of 96%, between the values measured manually by ABS experts and those produced by Svetlana classifier. For this application, Svetlana is a powerful ally for medium throughput screening. It makes it possible to quantify the effects of various drugs with a good accuracy and with a remarkably small need for human interactions.

## Discussion

### Efficiency

Svetlana requires little computational power, time resources, training data and it uses minimalist CNN architectures. We discuss these features below in more detail.

**Power consumption** Table 1 summarizes the computational cost for three different training tasks. Computing the energy footprint is a complex task^22^ and the numbers below are indicative. It is computed using the thermal design power (TDP) given by the manufacturer. On a regular GPU card, we see that the cost is proportional to the training time and not higher than working on a laptop for a few hours.

**Figure 4:**
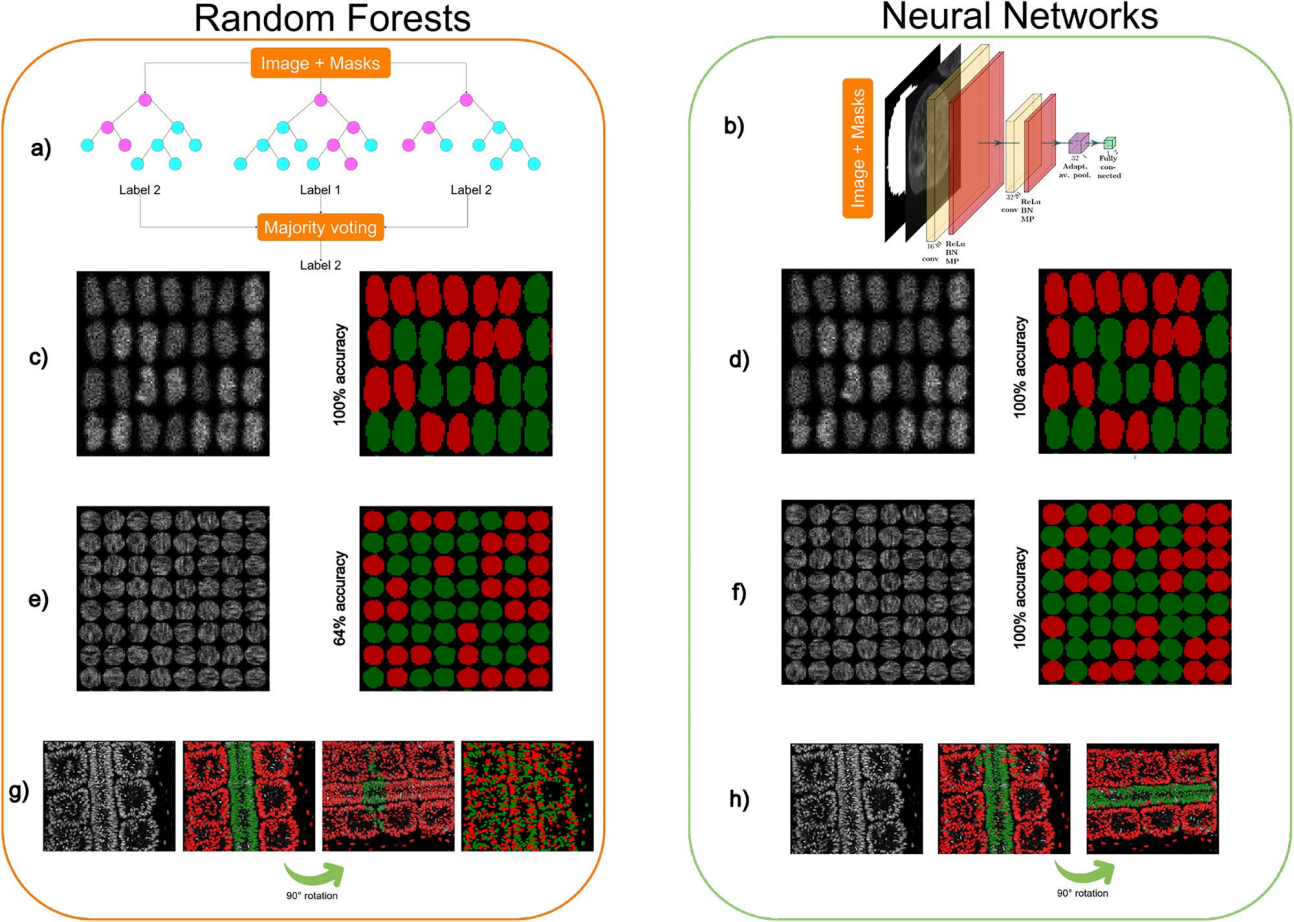
Comparing random forests and neural networks—(**a**) The principle of random forest classifiers: a few (10–1000) random decision trees are constructed and a majority voting allows making a prediction. (**b**) A convolutional neural network classifier makes a prediction by a sequence of convolutions, nonlinear activation functions and pooling. (**c–h**) Various classification tasks performed by each classifier. In order to facilitate the visualization, only small portions of the images are displayed. (**c,d**) Synthetic cell classification with differences of average gray level. Both Ilastik and a simple neural network yields 100% accuracy with as little as 10 labels. (**e–f**) Anisotropic texture classification. The two textures possess the same mean and variance, but different orientations. The random forest yields unsatisfactory results (64%) whatever the number of labels. The neural network yields 100% accuracy with only 45 labels. The reason for the failure of the random forest is that no pre-defined feature allows discriminating the texture orientations. On their side, neural networks are able to learn the right features with just a few annotations. (**g–h**) 2D slice of a two-photon microscope image of a quail embryo^9^ (courtesy of B. Bénazéraf). It shows a neural tube surrounded by somites. (**g**) From left to right: the slice containing 854 nuclei—the classification result using 35 labels. Thanks to the use of spatialization parameters, Ilastik provides excellent results for this task. However, if the classifier is applied to the rotated image, it yields unsatisfactory results since the spatialization changed. If the spatialization features are removed to construct the decision trees, Ilastik fails to classify the cells. (**h**) Svetlana result with 169 labels: it leads to a few mis-classifications on the original image. Nevertheless, it remains effective when the image is rotated thanks to the use of data augmentation during the training. This experiment overall shows the advantages of learning the classification features and to use data augmentation to add desirable properties such as rotation invariance.

- CPU is a 1.9 GHz Intel Xeon in a desktop (TDP: 165W).
- GPU1 is a Quadro T2000 with 4 GB RAM in a laptop (TDP: 40W).
- GPU2 is an RTX4000 with 8 GB RAM in a desktop (TDP: 160W).
- Dataset 1 is the oriented textures presented in Fig. 4e (45 labels).
- Dataset 2 is the 3D neural tube image presented in Fig. 1a (169 labels).
- Dataset 3 is the osteoclast dataset presented in Fig. 3 (5400 labels).

**Table 1:** Computation time and energy consumption in watt-hour (Wh) or kilowatt-hour (KWh) for different hardware configurations.

	Dataset 1	Dataset 2	Dataset 3
CPU	10 s–0.5 Wh	6 h 15–1 kWh	5.8 days–23 kWh
GPU1	8.5 s–0.1 Wh	6 h–0.24 kWh	26 h–1 kWh
GPU2	4.3 s–0.2 Wh	45’–0.1 kWh	18 h–2.9 kWh

**Towards small data?** Training a neural network classifier with just a few annotations (10-1000) in a few seconds goes against conventional wisdom. Indeed, complex architectures are usually trained for days or weeks with huge datasets. For instance, the data science bowl used to train the CellPose models contains 37,333 segmented nuclei in 841 2D images from more than 30 different imaging modalities. It is therefore legitimate to question whether the training phase could yield a good classifier. In practice, it turns out that this approach provides remarkable results with an accuracy sometimes higher than $90\%$ for complex tasks. This may not be on par with the best possible results, but still sufficient for many quantitative analyses in biology.

Let us mention that a few recent works point out that training with a minimal amount of data and *overparameterized* networks is a rich research avenue^23^. This is exactly the setting explored in Svetlana. As far as we know, complete theoretical explanations are still lacking. One possible way to interpret this is Ockham razor’s principle. The neural network architecture together with the training algorithm favor the “simplest” answer capable of explaining the observations. In our experiments, elementary networks tend to perform better than more complicated ones with many parameters. This looks quite natural, since a neural network with few parameters limits the expressiveness of the classifier and acts as a regularizer for the problem.

When high accuracy is critical, it is very likely that neural networks trained on large datasets would perform better. Unfortunately, such datasets are usually just not accessible. Each biology laboratory explores different tissues, at a different scale with a different modality and focus. Each collected image and labeling can be costly both in terms of money, know-how and time. Hence, Svetlana covers a crucial need which seems yet unmet. In addition, if general purpose classifiers appears in the future, they could be easily integrated and retrained within Svetlana.

**Lightweight models** Choosing the best neural network architecture is a time consuming art, not accessible to non-experienced users. A method that would provide completely different scores depending on the architecture is therefore not acceptable. Hopefully, it turns out that all the minimalist architectures we designed provide fairly similar classification scores. To illustrate this fact, we studied the influence of the architecture in terms of the depth and width parameter for the 3D experiment on the developing quail embryo. Table 2 displays the accuracy scores for every architecture. As can be seen here, all of them provide a fairly similar accuracy. Svetlana makes it easy to test a few different architectures, and visually compare the results. Overall, this experiment reveals that working with a single architecture (e.g. depth 3, width 32) provides consistently good classification results.

**Table 2:** Classification accuracy for the quail embryo classification problem, depending on the neural network architecture. In this experiment, we varied the depth and width of the proposed custom 3D convolutional network. All the models have been trained for 600 epochs. The columns acc. 1 and acc. 2 represent the classification accuracy for the neural tube and somites nuclei respectively. They are sorted from the most to the least accurate. As can be seen, the classification rate depends only mildly on the architecture defined through its width and depth.

Depth	Width	Acc. 1	Acc. 2	Avg acc.
3	32	0,946	0,938	0.9419
3	64	0.921	0.959	0.9402
2	16	0.938	0.937	0.9374
3	16	0.916	0.948	0.9318
2	64	0.917	0.94	0.9304
2	8	0.906	0.951	0.928
2	32	0.914	0.936	0.9253
3	8	0.90	0.935	0.9170
3	4	0.865	0.961	0.9131
2	4	0.884	0.936	0.9100
3	2	0.894	0.91	0.9010
2	2	0.834	0.925	0.8795

### CNN or random forests?

To the best of our knowledge, the software package offering the closest functionality to Svetlana is Ilastik^2^, and especially its *object classification* module. It is an excellent and powerful tool, which is widely adopted in biology laboratories. Despite obvious similarities, the backbones of the two applications are fundamentally different: Ilastik’s classifier is currently based on random forests^24^ while Svetlana’s relies on neural networks.

Random forests require a *pre-defined set of features*, such as geometrical properties of the segmentation mask (volume, diameter, location, ...), or intensity properties inside and outside the segmented zone (mean intensity, variance, quantile, ...). These features are then assembled to construct a set of random decision trees. This process can be achieved through many different randomized techniques^25^. The final decision taken by the random forest classifier is based on a majority vote using the output of each tree, see Fig. 4a.

Deep neural networks and especially the convolutional neural networks used in this paper (see Fig. 4b) work very differently. One of their main difference is that they are able to infer *automatically* the features, instead of having a fixed set of pre-defined image characteristics. The features (here, convolution filters and biases) are learned automatically from the labeled data using stochastic gradient descents.

Both approaches have their pros and cons. Random forests are typically easier to interpret than neural networks since their features have a clear meaning right from the start. If the features are discriminant, they can perform extremely well with few training examples and little risk of over-fitting, see Fig. 4c and d.

These assets can also turn to disadvantages for more complex tasks. It is indeed possible that no feature enables discriminating different categories, see Fig. 4e and f. On their side, neural networks are able to learn the features automatically and therefore perform more diverse and complex tasks than random forests. Overall neural networks tend to perform better, see for instance this comparison. However, this performance may require more training data, especially for large networks. This problem can be mitigated by choosing minimalist architectures when little training data is available or when the classification task is simple.

Finally, we illustrate a problem of *confounding variable* in Fig. 4g. In this experiment, we attempt to distinguish neural tube nuclei from somites nuclei in a developing quail embryo^9^. In Fig. 4g, the random forest classifier learned to distinguish the neural tube nuclei using their position in space rather than morphological or radiometric features. Hence, if the image is rotated, the classification becomes catastrophic. Similar issues may actually happen with convolutional neural networks. This is why the data augmentation mecanisms easily accessible in Svetlana are particularly important. In Fig. 4h, we added random rotations during the training phase to obtain a near rotation invariant classifier.

### Results interpretability

Aside from the classification results, another output of Svetlana is a trained neural network. Interpreting this algorithm, i.e., understanding how the decision process was made is a complicated task with many open research avenues. Answering recurrent questions such as: “What makes these populations different? Is it a difference of intensity, volume, ellipticity, or other things I may have missed?” is not directly accessible. Without further analysis, the neural network can therefore be considered as a black-box model, which is unquestionably a limitation of the approach. However, it is important to note that many alternative artificial intelligence models (including linear models or random forests) are often not easier to interpret^26^. In addition, the sole fact to know the existence of discriminating features significantly eases their determination.

That is why the development of interpretation tools has been an active area of research for some years now^27^. Algorithms Grad-CAM^12^ or guided Grad-CAM^28^ give the user an insight into the areas of the image that are prevalent in the classifier decision. We chose to integrate them in the prediction module of Svetlana, based on their popularity. New routines might be added in the future.

In addition to Grad-CAM, Svetlana offers the possibility to explore simple hypotheses by using data augmentation. Assessing whether the cell volume plays a role can be determined by adding dilations or not at the training time, and comparing the classification scores. The same experiment can be conducted using different types of intensity normalization, color changes,... to assess which features are discriminant. This is a simple approach to post hoc interpretation.

### Conclusion

We presented the main features and explained the functioning of a new plugin called Svetlana for the classification of segmentation results within the Napari environment. We showed through a few applications that Svetlana is a handful tool for challenging cell classification tasks. Neural networks are at the core of the program. To the best of our knowledge, Svetlana is the first open-source plugin offering such a large variety of learning mecanisms and tackles problems that would not be solvable with more elementary artificial intelligence tools such as random forests. Svetlana is open-source with a modular architecture allowing to integrate further features, following the most promising technological progress and user feedback. Through this paper, we wish to advertise this tool which should prove valuable to the biomedical community and beyond.

## Data Availability

The synthetic texture image and the 3D quail embryo one are directly accessible from the plugin as test images. Also, they are freely available on Zenodo at the following url: https://zenodo.org/records/7999871 and https://zenodo.org/records/7999871. PanNuke dataset is also open access. All the other datasets used and/or analysed during the current study are available from the corresponding author on reasonable request.

## Code availability

The source code is provided on BitBucket (https://bitbucket.org/koopa31/napari_svetlana/src/main/). A documentation is available at: https://svetlana-documentation.readthedocs.io/en/latest/. The plugin homepage is available at: https://www.napari-hub.org/plugins/napari-svetlana.

## References

[1] Graham S, Vu QD, Raza SEA, Azam A, Tsang YW, Kwak JT, Rajpoot N (2019). Hover-net: Simultaneous segmentation and classification of nuclei in multi-tissue histology images. Med. Image Anal..

[2] Berg S, Kutra D, Kroeger T, Straehle CN, Kausler BX, Haubold C, Schiegg M, Ales J, Beier T, Rudy M (2019). Ilastik: Interactive machine learning for (bio) image analysis. Nat. Methods.

[3] Stringer C, Wang T, Michaelos M, Pachitariu M (2021). Cellpose: A generalist algorithm for cellular segmentation. Nat. Methods.

[4] Cutler KJ, Stringer C, Lo TW, Rappez L, Stroustrup N, Brook Peterson S, Wiggins PA, Mougous JD (2022). Omnipose: A high-precision morphology-independent solution for bacterial cell segmentation. Nat. Methods.

[5] Fazeli, E., Roy, N. H., Follain, G., Laine, R. F., von Chamier, L., Hänninen, P. E., Eriksson, J. E., Tinevez, J.-Y., & Jacquemet, G. Automated cell tracking using StarDist and TrackMate. *F1000Research* **9**, (2020). 10.12688/f1000research.27019.1 PMC7670479 33224481

[6] von Chamier L, Laine RF, Jukkala J, Spahn C, Krentzel D, Nehme E, Lerche M, Hernández-Pérez S, Mattila PK, Karinou E (2021). Democratising deep learning for microscopy with zerocostdl4mic. Nat. Commun..

[7] Gómez-de Mariscal E, García-López-de Haro C, Ouyang W, Donati L, Lundberg E, Unser M, Muñoz-Barrutia A, Sage D (2021). DeepImageJ: A user-friendly environment to run deep learning models in ImageJ. Nat. Methods.

[8] Cazorla, C., Munier, N., Morin, R., & Weiss, P. Sketchpose: Learning to Segment Cells with Partial Annotations. Working paper or preprint (2023).

[9] Bénazéraf, B., Beaupeux, M., Tchernookov, M., Wallingford, A., Salisbury, T., Shirtz, A., Shirtz, A., Huss, D., Pourquié, O., François, P., & Lansford, R. Multiscale quantification of tissue behavior during amniote embryo axis elongation. *Development* (2017). 10.1242/dev.150557 PMC13471720 28835474

[10] Buslaev A, Iglovikov VI, Khvedchenya E, Parinov A, Druzhinin M, Kalinin AA (2020). Albumentations: Fast and flexible image augmentations. Information.

[11] Perkel JM (2021). Python power-up: New image tool visualizes complex data. Nature.

[12] Selvaraju, R. R., Cogswell, M., Das, A., Vedantam, R., Parikh, D., & Batra, D. Grad-cam: Visual explanations from deep networks via gradient-based localization. In *Proceedings of the IEEE International Conference on Computer Vision*, pages 618–626, (2017).

[13] Paszke, A., Gross, S., Massa, F., Lerer, A., Bradbury, J., Chanan, G., Killeen, T., Lin, Z., Gimelshein, N., Antiga, L., et al. Pytorch: An imperative style, high-performance deep learning library. *Adv. Neural Inform. Process. Syst.* **32**, (2019).

[14] Chiu C-L, Clack N (2022). napari: A python multi-dimensional image viewer platform for the research community. Microscopy Microanal..

[15] He, K., Zhang, X., Ren, S., & Sun, J. Deep residual learning for image recognition. In *2016 IEEE Conference on Computer Vision and Pattern Recognition (CVPR)*, pages 770–778 (2016).

[16] Huang, G., Liu, Z., Van Der Maaten, L., & Weinberger, K. Q. Densely connected convolutional networks. In *Proceedings of the IEEE Conference on Computer Vision and Pattern Recognition*, pages 4700–4708 (2017).

[17] Krizhevsky, A., Sutskever, I., & Hinton, G. E. Imagenet classification with deep convolutional neural networks. In F. Pereira, C. Burges, L. Bottou, and K. Weinberger, editors, *Advances in Neural Information Processing Systems, volume 25*. Curran Associates, Inc., (2012).

[18] Kingma, D. P., & Ba, J. Adam: A method for stochastic optimization. In *ICLR (Poster)*, (2015).

[19] Gamper, J., Koohbanani, N. A., Benes, K., Graham, S., Jahanifar, M., Khurram, S. A., Azam, A., Hewitt, K., & Rajpoot, N. Pannuke dataset extension, insights and baselines (2020). arXiv:2003.10778.

[20] Labour M-N, Riffault M, Christensen ST, Hoey DA (2016). Tgf$$\beta $$1-induced recruitment of human bone mesenchymal stem cells is mediated by the primary cilium in a smad3-dependent manner. Sci. Rep..

[21] Stringer, C. & Pachitariu, M. Cellpose 2.0: How to train your own model (2022). 10.1038/s41592-022-01663-4 PMC9718665 36344832

[22] Bouza L, Bugeau A, Lannelongue L (2023). How to estimate carbon footprint when training deep learning models? A guide and review. Environ. Res. Commun..

[23] Belkin M (2021). Fit without fear: Remarkable mathematical phenomena of deep learning through the prism of interpolation. Acta Numer.

[24] Breiman L (2001). Random forests. Mach. Learn..

[25] Biau G, Scornet E (2016). A random forest guided tour. TEST.

[26] Lipton ZC (2018). The mythos of model interpretability: In machine learning, the concept of interpretability is both important and slippery. Queue.

[27] Arrieta AB, Díaz-Rodríguez N, Del Ser J, Bennetot A, Tabik S, Barbado A, García S, Gil-López S, Molina D, Benjamins R (2020). Explainable artificial intelligence (xai): Concepts, taxonomies, opportunities and challenges toward responsible ai. Inform. Fusion.

[28] Springenberg, J. T., Dosovitskiy, A., Brox, T., & Riedmiller, M. Striving for simplicity: The all convolutional net (2014). arXiv:1412.6806.

